# Care of vision and ocular health in diabetic members of a national diabetes organization: A cross-sectional study

**DOI:** 10.1186/1472-6963-8-159

**Published:** 2008-07-28

**Authors:** Vibeke Sundling, Pål Gulbrandsen, Jak Jervell, Jørund Straand

**Affiliations:** 1 Department of Optometry and Visual Science, Buskerud University College, Kongsberg, Norway; 2 HØKH Research Centre, Akershus University Hospital and Faculty Division Akershus University Hospital, University of Oslo, Oslo, Norway; 3 Professor Emeritus, Bygdøy alle 25 A, 0262 Oslo, Norway; 4 Institute of General Practice and Community Medicine, University of Oslo, Oslo, Norway

## Abstract

**Background:**

Regular examination and early treatment of diabetic retinopathy can prevent visual loss. The aim of the study was to describe the care of vision and ocular health in people with diabetes in Norway.

**Methods:**

A cross-sectional questionnaire survey of a random sample (n = 1,887) of the Norwegian Diabetic Associations' (NDA) members was carried out in 2005. Questions were asked about care of vision and ocular health, history of ocular disease and visual symptoms, general medical history and diabetes management. The study was approved by the Regional Committee for Medical Research Ethics.

**Results:**

The response rate was 74%. Forty-four questionnaires with incomplete data regarding gender, age or type of diabetes were excluded, leaving 1352 cases (52% females) for analysis. 451 (33%) had type 1 and 901 (67%) had type 2 diabetes, the mean duration of diabetes was respectively, 22 (sd ± 14) and 10 (sd ± 9) years. In all 1,052 (78%) had their eyes examined according to guidelines and 1,169 (87%) confirmed to have received information about regular eye examinations. One in two recalled to have received such information from their general practitioner. To have received information about the importance of eye examinations (PR 3.1, 95% CI 2.4 to 4.0), and diabetes duration > 10 years (PR 1.2, 95% CI 1.2 to 1.3), were independently associated with reporting regular eye examinations. A history of diabetic retinopathy was reported by 178 (13%) responders, of which 101 (57%) reported a history of laser treatment. Responders who had regular eye examinations reported more frequently a history of diabetic retinopathy (19% vs. 5%, p < 0.001). The frequency of retinopathy was significantly higher in responders with reported HbA1c values above treatment target (23% vs. 13%, p = 0.001). However, in responders who were not regularly examined, there was no difference in reported frequency of retinopathy with regard to HbA1c level.

**Conclusion:**

Eight out of ten diabetic members of the NDA had their eyes examined according to current guidelines and the majority was well informed about the risk of vision loss due to diabetes. The results indicate that the reported history of diabetic retinopathy likely underestimates the prevalence of retinopathy.

## Background

In Western societies, diabetic retinopathy is one of the leading causes of visual impairment and blindness in the working age group [1-3]. The prevalence of diabetic retinopathy in Norway is sparsely described in the literature [4,5]. It is estimated that 90–120,000 Norwegians have known diabetes and that probably just as many have undiagnosed diabetes [6]. Most diabetic patients will develop some degree of retinopathy. Studies indicate that between 6% and 30% will develop sight threatening retinopathy during the course of their illness [7-14]. In Western Europe, diabetic retinopathy accounts for 4.7–13.3% of the blind and partial sight registrations [3,15-17]. Regular examination of ocular health and early treatment of diabetic retinopathy can prevent most cases of visual loss [18-22], and ophthalmologic screening of patients with diabetes is more cost-effective than many other health interventions for detecting and treating disease [21,23]. The Norwegian College of General Practitioners has published guidelines for examination of ocular health in patients with diabetes, first issued in 1988 [24]. Table 1 shows the Norwegian practice guidelines compared to practice guidelines in the United Kingdom, USA and Australia. In 1996 only 53% of diabetic patients seen in general practice were managed according to these guidelines [25,26].

**Table 1:** Practice guidelines for HbA1c treatment target and management of ocular health in patients with diabetes.

	Norway*	**United Kingdom**^†^	**USA**^‡^	**Australia**^§^
**HbA1c treatment target**				
Children/Type 1 < 18 years		< 7.5%	< 7%	
Type 1 > 18 years		< 7.5%	< 7%	
Type 2		6.5–7.5%	< 7%	
Younger (<80 years)	< 7%		< 7%	
Older (>80 years)	< 9%		< 7%	
**Screening for diabetic retinopathy**				
**First examination**				
Children/Type 1<18 years		At age 12 years		At puberty
< 30 years/Type 1	5 years after diagnosis	At diagnosis	5 years after diagnosis	At diagnosis
> 30 years/Type 2	At diagnosis	At diagnosis	At diagnosis	At diagnosis
**Follow-up in absence of retinopathy**				
Children/Type 1 < 18 years		Annually	Annually	At least biannually
< 30 years/Type 1	Annually	Annually	Annually	At least biannually
> 30 years/Type 2	Annually/Biannually	Annually	Annually	At least biannually
**Follow-up in retinopathy**	Individual	Individual	Individual	Individual

Norwegian practice guidelines compared to practice guidelines in the United Kingdom, USA and Australia at the time (2005) of the study.

*The Norwegian College of General Practitioners, ^†^National Institute for Clinical Excellence, ^‡^American Diabetes Association, ^§^National Health and Medical Research Council

The aim of this study was to describe and analyse the care of vision and ocular health among people with diabetes in Norway. Secondly, we wanted to explore their sources of information regarding ocular care. Finally we liked to assess the reported care in relation to established practice guidelines and to identify features associated with good practice.

## Methods

The study had a cross-sectional design. A random sample of persons with diabetes, drawn from the member list of the Norwegian Diabetes Association (NDA), was invited to participate in a questionnaire survey. At the time of the study, NDA was the only national registry of adults with diabetes. NDA is a voluntary, independent organization with the objective of serving people with diabetes and others who have an interest in diabetes. In 2005 NDA had 35,058 members, including mainly people with diabetes, some of their relatives (about 900), and around 3,000 health care professionals. The type of diabetes was not recorded in the NDA membership registry. A random sample comprising about 6% of NDA members 18 years and older was drawn by computer from the NDA membership registry. They were subsequently sent a postal questionnaire in October-December 2005. Non-diabetics, deceased members and members with unknown address or living abroad were excluded, leaving an eligible sample of 1,887. Information about the study, the voluntary nature of participation, confidentiality and study approval were given on the front page of the questionnaire and the return of a completed questionnaire was regarded as written consent. Reminders were sent once to all participants. The questionnaire had been assessed in a pilot survey. This pilot survey led to the inclusion of a question regarding source of information about the importance of regular eye examinations. The questionnaire included questions about care of vision and ocular health, history of ocular disease and visual symptoms, as well as details about type of diabetes, year of diagnosis, treatment, blood glucose stability, most recently recorded HbA1c and blood pressure, antihypertensive and cholesterol lowering medication, and current and previous smoking. In the questionnaire, regular examination was defined as examination at regular intervals, e.g. yearly, every six months or monthly. Furthermore, eye examination and vision examination was defined as examination of the back of the eye/retina and examination of sight, respectively. The specific questions on eye examination were: "Do you have your eyes regularly examined due to your diabetes?" and "In general, how long time is it between the eye examinations?".

In 2001 a list system was implemented in Norwegian general medical practice, implying that all citizens were listed with one particular general practitioner (GP). In this system the GP has the primary responsibility for the management and follow-up of patients with diabetes. Referral to a specialist (ophthalmologist) must be made by a GP. The Norwegian College of General Practitioners guidelines [26] were used as standard of patient care for comparison with reported care (Table 1).

Data analysis was performed with the statistical package SPSS version 13.0. Questionnaires with missing data for gender, age or type of diabetes, or diabetes other than type 1 and type 2 (3%) were excluded from analysis. The data were analysed in frequency and summation tables; group differences were analysed using student-t, chi-square and Fisher's exact tests. A p-value < 0.01 was considered statistically significant. Features associated with known history of diabetic retinopathy, visual symptoms, regular follow up and lack of eye examination were analysed by univariate and multiple logistic regression. Variables with p ≤ 0.25 from the univariate analyses were entered into the logistic regression models. Additionally, the prevalence rate ratios (PR) were calculated for features associated with regular eye examination and history of diabetic retinopathy to provide a natural intelligible effect measure and to allow for comparison to the prevalence odds ratios (POR).

Data were collected anonymously and the study was approved by the Regional Committee for Medical Research Ethics.

## Results

The total number of responders was 1,396 (74%). Forty-four questionnaires had missing information regarding either age, gender, type of diabetes or diabetes other than type 1 and type 2. The study is based on the remaining 1,352 cases, 699 (52%) were females. In all, 451 (33%) responders had type 1 diabetes and 901 (67%) had type 2 diabetes. Table 2 shows basic demographic and medical data of the responders.

**Table 2:** Demographic and medical characteristics of responders with type 1 and type 2 diabetes (n = 1,352), n (%)

	**All patients ** **(n = 1,352)**		**Type 1 ** **(n = 451)**		**Type 2 ** **(n = 901)**	
**Sex distribution**						
Female	699	(51.7)	247	(54.8)	452	(50.2)
Male	653	(48.3)	204	(45.2)	449	(49.8)
Age distribution*						
< 20 years	7	(0.5)	6	(1.3)	1	(0.1)
21–30 years	55	(4.1)	54	(12.0)	1	(0.1)
31–40 years	108	(8.0)	87	(19.3)	21	(2.3)
41–50 years	185	(13.7)	106	(23.5)	79	(8.8)
51–60 years	315	(23.3)	91	(20.2)	224	(24.9)
61–70 years	349	(25.8)	71	(15.7)	278	(30.9)
71–80 years	261	(19.3)	30	(6.7)	231	(25.6)
81–90 years	72	(5.3)	6	(1.3)	66	(7.3)
**Mean age (sd)**	59	(± 15)	48	(± 15)	64	(± 11)
**Mean duration of diabetes (sd)**^‡^	14	(± 12)	22	(± 14)	10	(± 9)
**Mean HbA1c at last diabetes follow up (sd)**^§^	7.3	(± 1.2)	7.5	(± 1.0)	7.1	(± 1.3)
HbA1c within guideline treatment target*^,§^						
All patients (HbA1c <7%/<9% depending on age)	461	(41.2)	107	(26.4)	354	(49.6)
Patients = 80 years (HbA1c <7%)	420	(39.1)	103	(25.6)	317	(47.2)
Patients > 80 years (HbA1c <9%)	41	(87.2)	4	(100)	37	(86.0)
**Stable blood glucose level previous year**^∥^	845	(63.9)	258	(58.6)	587	(66.6)
Diabetes treatment*						
Diet (n = 757)	668	(88.2)	129	(66.2)	539	(95.9)
Exercise (n = 657)	536	(81.6)	112	(59.6)	424	(90.4)
Weight reduction (n = 430)	197	(45.8)	16	(10.4)	181	(65.6)
Oral medication (n = 771)	564	(73.2)	16	(10.3)	548	(89.1)
Insulin (n = 896)	742	(82.8)	443	(99.8)	299	(66.2)
Blood pressure and cholesterol medication*						
Blood pressure (n = 1,337)	687	(51.4)	142	(31.6)	545	(61.4)
Cholesterol (n = 1,324)	591	(44.6)	123	(27.7)	468	(53.2)
**Smoking**						
Present smoker^† ^(n = 1,345)	203	(15.1)	88	(19.6)	115	(12.8)
Previous smoker (n = 1,306)	716	(54.8)	216	(50.2)	500	(57.1)
**Known history of ocular disease**						
Cataract* (n = 1,019)	261	(25.6)	63	(17.2)	198	(30.4)
Diabetic retinopathy* (n = 1,058)	182	(17.2)	91	(23.3)	91	(13.6)
Glaucoma* (n = 905)	93	(10.3)	14	(4.2)	79	(13.8)
Age-related macula degeneration^† ^(n = 857)	35	(4.1)	6	(1.9)	29	(5.4)
Hypertensive/occlusive vascular retinopathy (n = 851)	19	(2.2)	3	(0.9)	16	(3.0)
**Laser treated diabetes related ocular disease**^¶^						
History of diabetic retinopathy reported	105	(10.0)	57	(14.7)	48	(7.2)
History of diabetic retinopathy not reported*	19	(1.8)	7	(1.8)	12	(1.8)

Pearson chi-square *p < 0.001 and ^†^p < 0.01 between type 1 and type 2 diabetics.

Data missing for ^‡^31,^§ ^232, ^∥^30 and ^¶^300 responders

In all, 1,141 (85%) of the responders had their eyes regularly examined. Of these 1,052 (92%) were examined according to recommended follow up schedule. Only 6% reported never to have had their eyes examined. In persons with type 1 diabetes, 88% (358/407) were examined annually or more frequently: respectively 2%, 15%, 3% and 69% were examined every 1–3 months, 4–6 months, 7–9 months and 10–12 months. In persons with type 2 diabetes, 98% (694/711) were examined biannually or more frequently: respectively 2%, 13%, 1%, 63% and 18% were examined every 1–3 months, 4–6 months, 7–9 months, 10–12 months and 12–24 months. The time of the first examination was in accordance to guidelines in 31% of responders with type 1 diabetes and in 47% of responders with type 2 diabetes (Table 3). For all responders, the median interval between eye examinations was 12 months (65%). Eight-teen percent were regularly examined more frequently. In total, 1,169 (87%) responders confirmed to have received some information about the importance of having their eyes regularly examined due to their diabetes. Responders who had their eyes examined according to guidelines were more than twice as likely to have received such information than responders who did not undergo regular eye examinations (95% vs. 42%, p < 0.001). Having received information about the importance of eye examinations, and diabetes duration of more than 10 years, were both independently associated with regular eye examinations (Table 4).

**Table 3:** Information about eye examination and frequency of eye and vision examination (n = 1,352), n (%)

	**All patients ** **(n = 1,352)**		**Type 1** ** (n = 451)**		**Type 2** ** (n = 901)**	
Informed about the importance of eye examination*/^‡,§^	**1,169**	**(86.8)**	**433**	**(96.2)**	**736**	**(82.1)**
Source of information not mutually exclusive*						
General practitioner^§^	678	(58.0)	205	(47.3)	473	(64.3)
Ophthalmologist	515	(44.1)	202	(46.7)	313	(42.5)
Hospital^§^	338	(28.9)	224	(51.7)	114	(15.5)
Other medical practitioner^§^	114	(9.8)	95	(21.9)	19	(2.6)
Optometrist	93	(8.0)	41	(9.5)	52	(7.1)
Leaflets/Journal of the Norwegian Diabetes Association^∥,§^	298	(25.5)	136	(31.4)	162	(22.0)
Diabetes patient education course	218	(18.6)	77	(17.8)	218	(18.6)
Media	68	(5.8)	24	(5.5)	44	(6.0)
Other persons with diabetes^§^	94	(8.0)	47	(10.9)	47	(6.4)
**First eye examination after diagnosis**^†,§^						
Within 1 year	538	(40.3)	121	(26.9)	417	(47.1)
Within 1–5 years	433	(32.5)	138	(30.7)	295	(33.3)
After more than 5 years	221	(16.6)	140	(31.2)	81	(9.2)
Never examined	74	(5.5)	6	(1.3)	68	(7.7)
Unsure	68	(5.1)	44	(9.8)	24	(2.7)
**Regular eye examination reported by one or more methods**^‡,§^						
Eye examination by one or more methods	1,141	(85.4)	416	(92.7)	725	(81.7)
Examination by ophthalmologist	965	(84.6)	339	(81.5)	626	(86.3)
Fundusphotography	443	(38.8)	202	(48.6)	241	(33.2)
Examination by optometrist	90	(7.9)	30	(7.2)	60	(8.3)
Examination by general practitioner	21	(1.8)	6	(1.4)	15	(2.1)
**Regular vision examination reported by one or more methods**						
by one or more methods	1,161	(85.9)	386	(85.6)	775	(86.0)
by ophthalmologist	979	(84.3)	330	(85.5)	649	(83.7)
by optometrist	252	(21.7)	84	(21.8)	168	(21.7)
by other health care provider	33	(2.8)	18	(4.7)	15	(1.9)
by medical doctor	29	(2.5)	8	(2.1)	21	(2.7)

Missing data for *5,^†^18, ^‡^16 responders.

Pearson chi-square ^§ ^p < 0.001 between type 1 and type 2 diabetics.

^∥ ^Journal of the Norwegian Diabetes Association

**Table 4:** Characteristics associated with regular eye examination in patients with diabetes

**Characteristic (association)**	**Eye exam (%) in group with characteristic**	**Eye exam (%) in group without characteristic**	**Crude prevalence ratio (95% CI)**	**Crude odds ratio (95% CI)**	Adjusted* odds ratio (95% CI)	P value*
Information on eye examination	93.0	29.7	3.1 (2.4 to 4.0)	31.5 (20.7 to 48.1)	27.4 (16.7 to 44.8)	<0.001
Diabetes duration > 10 years	93.5	75.0	1.2 (1.2 to 1.3)	4.8 (3.4 to 6.8)	3.1 (2.0 to 5.1)	<0.001
Visual problems related to diabetes	97.4	84.5	1.6 (1.1 to 1.2)	6.9 (2.5 to 19.1)	3.6 (1.2 to 10.6)	0.024
Using one or more optical corrections	85.9	81.5	1.1 (1.0 to 1.1)	1.4 (0.5 to 1.1)	1.5 (0.8 to 2.9)	0.234
Type of diabetes (Type 1)	92.7	81.7	1.1 (1.1 to 1.2)	0.3 (0.2 to 0.5)	0.9 (0.5 to 1.7)	0.842

*Multivariate logistic regression analysis

Spectacles and/or contact lenses were used by 1,188 (88%) of the responders. The vision was regularly examined in 1,045 of the responders who used optical correction. This was significantly more frequent than among responders who did not use optical correction (88% vs. 71%, p < 0.001). However, there was no significant difference in the frequency of eye examinations between the groups. During the previous year, 611 (45%) of the responders had experienced some kind of visual problems. Nearly two in five reported to be helped by optical correction.

Visual problems due to diabetes were reported by 156 (12%) responders. A history of laser treatment of ocular disease related to diabetes was reported by 81% of the responders with visual problems related to diabetes and known history of diabetic retinopathy. In all, 178 (13%) reported a history of diabetic retinopathy, of these 101 (57%) also reported a history of laser treatment. Diabetic retinopathy was associated with type 2 diabetes, diabetes duration >10 years, use of oral anti-diabetic agents, use of insulin, HbA1c above 7%, unstable blood glucose levels and the use of anti-hypertensive medication. In a multivariate logistic regression analysis diabetes duration was the only reported factor independently associated with a history of diabetic retinopathy. The prevalence of diabetic retinopathy was higher in patients with diabetes duration longer than 10 years, than in patients with shorter disease duration prevalence ratio (95% CI) of 3.5 (2.5 to 5). Moreover, a history of diabetic retinopathy was more frequently reported by responders who had their eyes regularly examined (Table 5).

**Table 5:** History of diabetic retinopathy as reported by diabetic patients (n = 900) by blood glucose level* and eye examination, n (%)

	**Known history of diabetic retinopathy**		**No history of diabetic retinopathy**	
**Regular eye examination**^†^				
HbA1c within treatment target	39	(12.9)	264	(87.1)
HbA1c above treatment target	113	(23.3)	371	(76.7)
**No regular eye examination**				
HbA1c within treatment target	3	(5.5)	52	(94.5)
HbA1c above treatment target	3	(5.3)	54	(94.7)

*HbA1c in accordance with treatment target level given in the Norwegian College of General Practitioners' guidelines.

^† ^Pearson chi-square p = 0.001 between persons with HbA1c within and person with HbA1c above treatment target.

## Discussion

The vast majority of persons with diabetes responding to this survey had their eyes examined according to guidelines advised by the Norwegian College of General Practitioners [26], Table 1. Diabetes duration and having received information about potential eye complications were independently associated with eye care management according to the guidelines.

Compared to cross-sectional surveys of the general diabetic population in UK, Australia and the United States [27-30] the number of persons with diabetes who had their eyes examined according to guidelines was equal or higher in our study. Furthermore, the proportion who had been examined according to guidelines (78%) were noticeably higher than (53%) reported in a previous Norwegian survey from 1996 [25]. Our study may reflect an actual improvement in the management of ocular care in people with diabetes in Norway. Improved eye care in people with diabetes has also been reported in the United States (1988–2002) and Australia (2003–2005) [28,31]. Unpublished data from a study undertaken in Norwegian general practice in 1999/2000 revealed that three out of four patients with diabetes were managed according to current guidelines (Tor Claudi 2007, personal communication), indicating an improvement of care compared to 1996 [25]. The improved care could be explained by increased focus on diabetes as a modern epidemic, increased professional knowledge about clinical guidelines, increased patient knowledge, and the coverage of diabetes in mass media. However, the observation may also to some extent reflect selection; the responders in our study were members of the NDA and their rate of regular eye examination is probably higher than in the general diabetic population due to a higher interest in own health and more exposure to patient education materials. Moreover, the overrepresentation of persons with type 1 diabetes and persons with long term illness are probably other important explanations for the high compliance with the screening programme. Additionally, self-reports may overestimate the frequency of eye examinations due to recall bias, telescoping and social acceptance [32].

Utilization of eye care services is associated with the uses of health services in general, and with health promotion campaigns [27,29,31]. The fact that half of the patients had not received information about the importance of regular eye examinations by their GP and that not all patients had their eyes examined according to practice guidelines, suggest that there are still potentials for improving the quality of care. Almost 90% of the responders in our study used some form of optical correction and diabetic patients are frequently seen in Norwegian optometric practice [33]. This suggests a role for optometrists in promoting regular eye examinations in diabetes patients. Moreover, the quality of ocular care can probably be improved by strengthening the optometrist-GP communication ensuring that optometrists regularly inform the GP about significant ocular findings (e.g. retinopathy in patients with diabetes) and also about patients who are not regularly examined. This could be achieved through continuing education of Norwegian optometrists and professional awareness campaigns.

The prevalence of known history of diabetic retinopathy among the responders corresponds with the total prevalence of diabetic retinopathy reported in a small Norwegian community, the Eigersund study [4]. However, it is lower than the reported prevalence in recent Danish studies [34,35]. On the other hand, the rate of responders with a history of laser treatment of ocular complications due to diabetes (three out of five) corresponds well with the figures in the Danish studies. The Danish studies are based on clinical examination rather than on self-reports. If we assume corresponding criteria for laser treatment in Norway and Denmark, this suggests an underestimation of the prevalence of diabetic retinopathy in our sample, maybe due to lack of knowledge about the presence of non-sight-threatening diabetic retinopathy among responders.

Tight control of blood glucose and blood pressure reduces the risk of progression towards sight threatening disease [36] and visual outcome in patients with diabetes is related to regular eye examination [22]. In our study, the rate of established diabetic retinopathy was four times higher in responders who had their eyes regularly examined as compared to those who did not attend regular eye examinations. Among responders who had their eyes regularly examined, the frequency of retinopathy was nearly twice as high among those who reported HbA1c values above treatment target. In responders who did not have their eyes regularly examined, however, the reported frequency of retinopathy was the same for both patients with HbA1c above and within treatment target. This further adds to the assumption of a probable underestimation of diabetic retinopathy in our population, not only due to lack of patient knowledge, but also due to lack of regular eye examinations.

The relatively high response rate implies that our findings are representative for diabetes members of the NDA. An important limitation is that the findings from this NDA membership survey cannot be directly extrapolated to the general diabetes population in Norway. The probable underestimation of diabetic retinopathy prevalence even in this patient group, may suggest that the underrecognition may be even larger in the general diabetes population. However, we did not verify whether the reported eye examination and medical history corresponded with medical records.

## Conclusion

The majority of diabetic members of NDA has their eyes examined according to existing guidelines and are well aware of the risk of vision loss due to diabetes. The reported prevalence of diabetic retinopathy is probably underestimated due to lack of knowledge about established retinopathy and undiagnosed diabetic retinopathy among the responders indicating a potential for improvement in care.

## Competing interests

The authors declare that they have no financial competing interests.

VS is a board member of the Norwegian Optometric Association and member of Norwegian Optometric Associations' board of continuing education.

## Authors' contributions

VS conceived of the study and participated in its design, acquired and statistically analysed the data and drafted the manuscript. PG helped designing the study, supervised the statistical and epidemiological analyses and scrutinized the manuscript for important intellectual content. JJ and JS participated in the design of the study and critically revised the manuscript. All authors read and approved the final manuscript.

## Pre-publication history

The pre-publication history for this paper can be accessed here:


